# Optimization of the molecular diagnosis of the acute *hepatitis E virus* infection

**DOI:** 10.1111/1751-7915.14247

**Published:** 2023-03-25

**Authors:** Pedro Lopez‐Lopez, Mario Frias, Ana Belén Perez‐Jimenez, Carolina Freyre‐Carrillo, Juan A. Pineda, Antonio Aguilera, Ana Fuentes, Juan Carlos Alados, Gabriel Reina, Encarnación Ramirez‐Arellano, Isabel Viciana, Joao Mesquita, Javier Caballero‐Gomez, Antonio Rivero‐Juarez, Antonio Rivero, for the HEPAVIR and GEHEP‐014 Study Groups

**Affiliations:** ^1^ Unit of Infectious Diseases, Hospital Universitario Reina Sofia, Instituto Maimonides de Investigación Biomédica de Córdoba (IMIBIC) Universidad de Córdoba (UCO) Cordoba Spain; ^2^ CIBERINFEC, ISCIII – CIBER de Enfermedades Infecciosas Instituto de Salud Carlos III Madrid Spain; ^3^ Clinical Microbiology Unit Hospital Universitario Reina Sofía, Instituto Maimónides de Investigación Biomédica de Córdoba (IMIBIC) Córdoba Spain; ^4^ Clinical Microbiology Unit University Hospital of Puerto Real Cádiz Spain; ^5^ Unit of Infectious Diseases and Microbiology. Hospital Universitario de Valme Seville Spain; ^6^ Microbiology Department Hospital Clínico Universitario & University of Santiago de Compostela (USC)/IDIS Santiago de Compostela Spain; ^7^ Clinical Microbiology Unit Hospital Universitario Clínico San Cecilio, Instituto de Investigacion Biosanitaria Ibs. Granada Spain; ^8^ Clinical Microbiology Unit Hospital Universitario de Jerez Cádiz Spain; ^9^ Microbiology Department Clínica Universidad de Navarra, ISTUN, Institute of Tropical Health, Universidad de Navarra, IdiSNA, Navarra Institute for Health Research Pamplona Spain; ^10^ Infectious Diseases, Microbiology and Preventive Medicine Unit, Department of Medicine Virgen Macarena Univ. Hospital, University of Sevilla/Biomedicine Institute of Sevilla Sevilla Spain; ^11^ Infectious Diseases, Microbiology and Preventive Medicine Unit Hospital Universitario Virgen de la Victoria Málaga Spain; ^12^ ICBAS – Instituto de Ciências Biomédicas Abel Salazar Universidade do Porto Porto Portugal; ^13^ Epidemiology Research Unit (EPIUnit) Instituto de Saúde Pública da Universidade do Porto Porto Portugal; ^14^ Laboratório para a Investigação Integrativa e Translacional em Saúde Populacional (ITR) Porto Portugal; ^15^ Departamento Sanidad Animal, Grupo de Investigación en Sanidad Animal y Zoonosis (GISAZ), UIC Zoonosis y Enfermedades Emergentes ENZOEM Universidad de Córdoba Córdoba Spain

## Abstract

To evaluate the diagnostic value of the combination of two broad‐range PCR assays targeting two different and conserved regions of the viral genome for the diagnosis of acute Hepatitis E virus (HEV) infection. Patients with acute hepatitis were prospectively recruited. In all, HEV‐IgM antibodies were tested together with evaluation of HEV viraemia by two PCR assays (ORF3 and ORF1). The number of individuals exhibiting negative IgM antibody results but carrying viral RNA was calculated by each PCR assay. Four‐hundred and seventy individuals were included, of whom 145 (30.8%) were diagnosed as having acute HEV. Of them, 122 (84.1%) exhibited HEV‐IgM antibodies, and 81 (55.8%) had detectable viral RNA for at least one PCR. Using the ORF3 molecular assay, 70 (48.3%) individuals were identified with HEV infection. When the ORF1 molecular assay was applied, 49 (33.8%) individuals were identified. The ORF3 assay detected viral RNA in 32 patients not detected by the ORF1 assay. In contrast, the ORF1 assay could amplify viral RNA in 11 patients who were not detected by the ORF3 assay. The parallel use of two broad‐range PCR assays significantly increased the performance of the molecular diagnosis of HEV.

## Graphical abstract

Our study optimize the molecular diagnosis of HEV using two PCRs assays. The combination of two targets significantly increase the diagnostic performance of HEV infection.

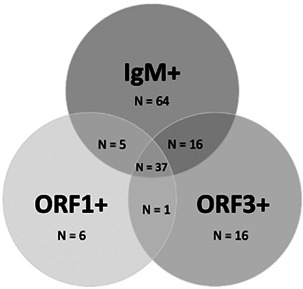

## INTRODUCTION

Hepatitis E virus (HEV), formally named *Paslahepevirus balayani*, is an RNA virus belonging to the *Hepeviridae* family (Orthohepevirinae subfamily) ranked as the main cause of acute viral hepatitis worldwide (WHO, 2020). The diagnosis of acute infection should be made by the combination of serum IgM anti‐HEV antibodies and viral RNA determination (European Association for the Study of the Liver, 2018). The combination of both markers is needed to increase the accuracy of the diagnosis (Rivero‐Juarez et al., 2021), because of the short duration of viraemia (approximately 15–20 days) and the delay between symptom onset and IgM conversion (Aggarwal, 2013). For this reason, the European Association for the Study of the Liver (EASL) clinical guidelines encourage the implementation of both techniques at the microbiological diagnosis level (European Association for the Study of the Liver, 2018). This approach could be optimized, only evaluating viral RNA in patients with non‐reactive IgM assays in whom clinical suspicion of HEV infection remains (Skittrall & Jalal, 2022).

Meanwhile, IgM determination is easy to implement, and the molecular diagnosis of HEV could be challenging because, although there are available different assays with a high accuracy for the amplification of HEV RNA, these suffer from different limitations that could challenge their diagnostic value. First, the sensitivity shown for endemic genotypes in Europe is low compared with other imported genotypes by several assays (Abravanel et al., 2013; Baylis et al., 2011, 2013). Second, the number of genotypes and subtypes described for HEV has increased to almost 50 (Casares‐Jimenez et al., 2021; Smith et al., 2020). Thus, these assays have not been evaluated for these new and emerging genotypes, and consequently, their sensitivity in this context is unknown. Finally, other assays are unable to detect properly the viruses included in the several genera of the Hepeviridae family, such as Rocahepevirus (Sridhar et al., 2022). *Rocahepevirus ratti* (RHEV) has shown to have zoonotic potential, describing cases of acute hepatitis in Asia and Europe in recent years (Rivero‐Juarez et al., 2022; Sridhar et al., 2021). Because this virus is present in rodents worldwide (Reuter et al., 2020), it is presumable that more zoonotic cases will be described in other countries and regions. For these reasons, the evaluation of an alternative approach to increase the performance of the molecular diagnosis of *Hepeviridae* family viruses may have important clinical value.

To increase the sensitivity and specificity of the molecular diagnosis of acute viral infection, different strategies have been applied. One approach is based on targeting two or more viral genome regions using the same (or different) PCRs. An example of this is the use of multiple targets (N, E, and RNA‐dependent RNA polymerase (RdRp) genes) for the diagnosis of SARS‐CoV‐2 infection (Tastanova et al., 2021), which has claimed to be a gold standard (Dramé et al., 2020). Another approach used is the combination of several probes in RT–PCR with a single‐nucleotide polymorphism. This approach has been described recently for HEV, allowing the simultaneous detection and typing of genotypes 1–4 (Ishida et al., 2022). There are several broad‐range PCR assays described and validated for the diagnosis of HEV virus, one allowing the detection of the entire HEV genotypes (Abravanel et al., 2012; Frías et al., 2021), and others permitting the detection of the majority of the *Hepeviridae* family strains (Drexler et al., 2012; Johne et al., 2010). Nevertheless, the combination of these broad‐range assays for the diagnosis of HEV infection has not been conducted. For this reason, the objective of our study was to evaluate the diagnostic value of the combination of two broad‐range PCR assays targeting two different and conserved regions of the viral genome of *Hepeviridae* family infection for the diagnosis of acute infection.

## EXPERIMENTAL PROCEDURES

### Ethical statement and approval of the study

This study was designed and conducted in accordance with the Declaration of Helsinki. The Ethics and Clinical Trials Committee (CEIC) of Andalucia (Spain) approved the study protocol, obtaining the informed consent of each patient (reference 4535). The SSPA Biobank has coordinated the collection, processing, handling and assignment of the biological samples used in this study in accordance with the standard procedures established for this purpose (agreement S2100110).

### Study population

For this analysis, we included patients prospectively recruited in a cohort composed of individuals with acute hepatitis diagnosed in nine hospitals from the Spanish National Health System between August 2019 and August 2022. For their inclusion in the cohort, patients should fulfil three criteria: (i) clinical and biological manifestations compatible with acute hepatitis, (ii) ALT level three times the upper limit of normal, and (iii) no etiological diagnosis after screening for hepatotropic virus infection, including serological and molecular markers for hepatitis A virus, hepatitis B virus, hepatitis C virus, cytomegalovirus, and Epstein–Barr virus. In all patients, ALT, AST, GGT, and bilirubin were measured.

### Evaluation of HEV serological and molecular markers

In all patients, a serum sample was analysed centrally for HEV serological and molecular markers in the Clinical Virology and Zoonoses lab of the Instituto Maimonides de Investigación Biomédica de Córdoba (IMIBIC). This analysis included IgM antibody determination and viral RNA evaluation.

HEV‐IgM antibodies were determined by enzyme immunoassay using the HEV‐IgM kit developed by Wantai (Beijing Wantai Biological Pharmacy Enterprise Ltd., Beijing, China) under an automated procedure (Triturus; Grifols), confirming those positive individuals by immunoblotting (recomLine HEV‐IgG/IgM; Mikrogen Diagnostik, Neuried, Germany).

The molecular analysis was conducted using two broad‐range methods allowing the detection of HEV and RHEV. For this, RNA was automatically extracted (QIAcube; Qiagen, Hilden, Germany) from 400 mL of serum using the QIAamp Mini Elute virus spin kit (Qiagen), where the RNA was eluted in 50 μL of RNA‐ase free water. For the molecular analysis, we applied two methods targeting two different regions of the viral genome. The first PCR assay (ORF3) was a real‐time quantitative PCR developed and validated by our group targeting a region of the ORF3 viral genome (Frías et al., 2021). The second PCR (ORF1) was developed and validated by Johne et al. for the detection of *Hepeviridae* strains and consists of a nested PCR targeting a conserved region of the viral polymerase located at ORF1 (Johne et al., 2010). For the first PCR (ORF3), the Qiagen one‐step PCR kit (Qiagen, Hilden, Germany) was used for 25 μL of the template (50 μL reaction volume). For the second PCR (ORF1), the same mix was used for the first round using a template of 10 μL (final Volume 50 μL), and Promega master Mix (Madison, USA) was used for the second round adding 5 μL of the first‐round product. The amplicons were examined on 1.5% agarose gels stained with RedSafeTM Nucleic Acid Staining solution. PCR products with the correct target size (approximately 334 nucleotides) were considered as positives after confirmation by both sense strand sequencing. As a positive control for all these reactions, the first World Health Organization International Standard for HEV RNA nucleic acid amplification test‐based assays, consistent with HEV genotype 3a and provided by the Paul‐Ehrlich‐Institut (PEI code 6219/10), was used. Primers and probe sets can be found in Supplementary Table S1.

The positive samples for any of the PCR assays employed were sequenced by nested RT–PCR targeting the ORF2 region according to a previously described procedure (Abravanel et al., 2012). Subtype assignment was performed using the HEVnet genotyping tool (https://www.rivm.nl/mpf/typingtool/hev/) and confirmed by BLAST analysis. Because this tool was updated on 1 September 2022, the genotype assignment was reconducted for the current analysis.

### Statistical analysis

The outcome variable was HEV or RHEV infection, defined as an individual exhibiting IgM antibodies and/or detectable viral RNA by at least one PCR method, according to the definitions of clinical guidelines. We showed the number of individuals identified as positive using both PCR assays in combination with IgM antibodies. The number of individuals exhibiting negative IgM antibody results but carrying viral RNA was calculated by each PCR assay. Finally, the characteristics of discordant individuals were described by each PCR assay.

## RESULTS

### Study population

The study population included 470 individuals suffering from acute hepatitis. Of them, 261 (55.5%) were male, with a median age of 49 years (IQR: 42–58 years). Regarding the liver function test, the median value for ALT was 198 U/L (IQR: 139–628 U/L); for AST, it was 100 U/L (IQR: 34–329 U/L); for GGT, it was 114 U/L (IQR: 59–332 U/L); and for bilirubin, it was 0.9 mg/dL (IQR: 0.5–3.1 mg/dL). Regarding comorbidities, 39 (8.2%) were people living with HIV, all but one with undetectable viral load. No other causes of underlying immunosuppression were found in the population.

### Screening for HEV infection

One hundred forty‐five individuals (30.8%) were diagnosed with acute HEV infection. Of them, 122 (84.1%) exhibited HEV‐IgM antibodies, and 81 (55.8%) had detectable viral RNA for at least one PCR. Among patients with a detectable viral load, the genotype could be obtained in 63 (77.7%). Of them, 60 were HEV (95.2%), and 3 were RHEV (4.8%). The three cases of RHEV were previously identified and reported (Rivero‐Juarez et al., 2022). Regarding HEV genotypes, 48 (80%) were genotype 3f, six (10%) genotypes were not assigned, four (6.6%) were genotype 3 m, one (2%) was genotype 3e, and the other (2%) was genotype 3 L (p). Fifteen of the 18 individuals for whom samples could not be sequenced had a viral load lower than 10,000 UI/mL, the limit of detection of the sequenced method employed. All the sequences are available in GenBank.

## PERFORMANCE OF DIAGNOSIS APPROACHES

Using the ORF3 molecular assay, 70 individuals were identified with HEV infection. This supposed the 48.2% of the total HEV cases diagnosed and the 86.4% of the patients with detectable viral RNA. Applying the ORF1 molecular assay, 49 individuals were identified, resulting in 33.7% of all cases with HEV infection and 60.4% of the patients with a detectable viral load.

**Figure 1:**
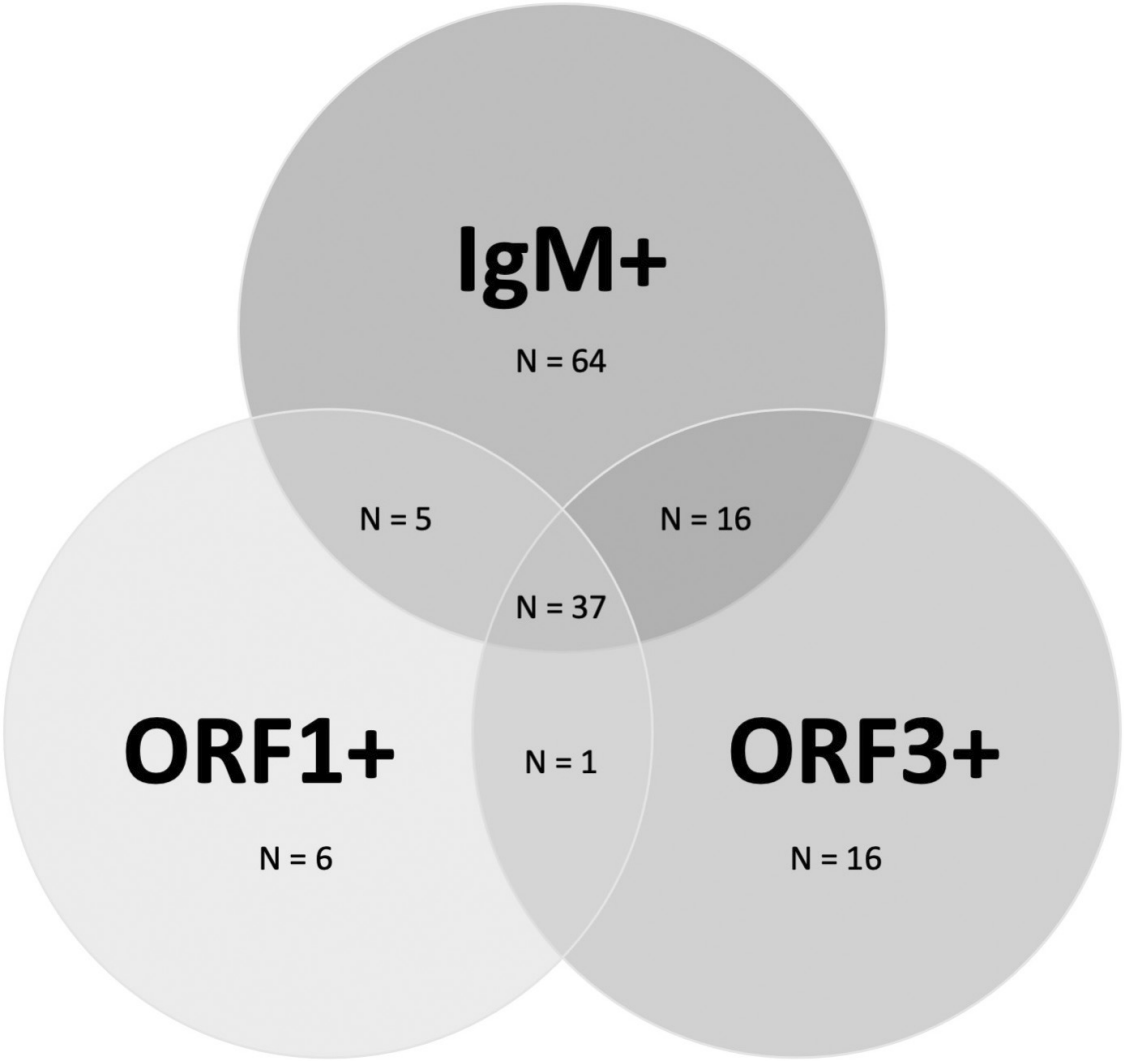
Venn diagram for the 145 patients with acute Hepatitis E based on IgM antibodies and two PCR assays.

In Figure 1, we show the Venn diagram based on these two molecular assays and IgM antibodies determination. The ORF3 assay detected viral RNA in 32 patients not detected by the ORF1 assay. In contrast, the ORF1 assay could amplify viral RNA in 11 patients who were not detected by the ORF3 assay.

Considering the IgM determination (Figure 1), the ORF3 assay was able to detect 17 individuals not detected by the antibody's determination or the ORF1 assay. In the same way, the ORF1 assay identified 7 positive individuals not diagnosed by IgM antibodies or the ORF3 assay. One patient was positive for both molecular assays but negative for IgM.

The combination of both assays allowed us to identify 23 patients with acute HEV or RHEV infection with respect to the single determination of IgM antibodies, supposing an increase in the diagnosis rate of 15.8%. Applying only the ORF3 or ORF1 assay, the increase in the diagnosis rate was 11% and 4.1%, respectively.

### Characteristics of discordant individuals

Of the 32 individuals identified with the ORF3 assay but not with ORF1, 17 (53.1%) had a viral load lower than 10,000 IU/mL (Table 1). Among those individuals whose sample could be sequenced, all carried genotype 3f (Table 1). Regarding patients in whom viral RNA was detected using the ORF1 assay but not ORF3 (Table 2), three were infected by RHEV, and the others were infected by HEV genotype 3f.

**TABLE 1:** Patients detected using the ORF3 PCR assay but not by the ORF1 PCR assay.

Patient	IU/mL	Genotype	GenBank ORF2	IgM
1	366	NS		Positive
2	528	NS		Positive
3	1876	NS		Positive
4	3533	NS		Positive
5	4186	NS		Positive
6	5754	3f	MT776554	Positive
7	13,653	3f	OL741644	Positive
8	16,030	3f	OL741635	Positive
9	16,825	NS		Positive
10	18,630	3f	OP121176	Positive
11	25,550	3f	MT250082	Positive
12	34,500	3f	MN628560	Positive
13	37,460	3f	OP121177	Positive
14	58,466	NS		Positive
15	177,638	3f	MN628562	Positive
16	420,000	3f	MN628563	Positive
17	2770	NS		Negative
18	340	NS		Negative
19	363	NS		Negative
20	874	NS		Negative
21	1170	NS		Negative
22	1907	NS		Negative
23	2350	NS		Negative
24	2859	NS		Negative
25	3633	NS		Negative
26	4409	NS		Negative
27	19,826	3f	OL741643	Negative
28	13,000	3f	MT776551	Negative
29	18,935	3f	OL741642	Negative
30	19,400	3f	MN628558	Negative
31	33,468	3f	MN628557	Negative
32	201,853	3f	MT776552	Negative

Abbreviations: IU/mL, International units per millilitre; NS, not sequenced; IgM, immunoglobulin M; ORF2, open reading frame 2.

**TABLE 2:** Patients detected using the broad‐range ORF1 assay but not by the ORF3 assay.

Patient	Genotype	GenBank ORF1	GenBank ORF2	IgM
1	3f	MN628559		Positive
2	3f	MN628565		Positive
3	*Rocahepevirus ratti*	OK082152		Positive
4	*Rocahepevirus ratti*	OK082153		Negative
5	*Rocahepevirus ratti*	OK082154		Negative
6	3f	OL741652		Negative
7	3f	OL741653		Positive
8	3f	OL741654	OL741645	Negative
9	3f	OL741655		Negative
10	3f	OP121174	OP121175	Positive
11	3f	OP177942		Negative

Abbreviations: ORF1, open reading frame 1; ORF2, open reading frame 2.

## DISCUSSION

Our study shows that the combination of two molecular assays targeting different conserved regions of the viral genome (such as viral capsid protein and RdRp protein) significantly increases the number of viraemic patients identified. Because of the HEV genome heterogeneity among and within genera, as well as genotype and subtype variability, the use of only one molecular assay could underestimate the number of individuals suffering from acute infection.

For this approach, we decided to evaluate the strategy of targeting two regions of the viral genome using two in‐house broad‐range molecular assays. We apply these two assays for two reasons. First, both assays have been validated for the identification of the most viral strains included in the *Hepeviridae* family (Frías et al., 2021; Johne et al., 2010). The ORF3 assay has been demonstrated to have a better performance than most commercial kits used for the diagnosis of HEV infection (Frías et al., 2021), and an assay targeting the ORF1 region has been shown to be useful for the identification of RHEV (Rivero‐Juarez et al., 2022). Second, because both reactions are available for the whole scientific community (including primers, probes and thermocycler conditions), a wide use of this approach is expected, especially focusing on labs from low‐income countries. Because HEV is considered as a neglected disease (Azman et al., 2019), and the majority of fatal outbreaks affect countries with very limited resources (Ahmed et al., 2022; Desai et al., 2022), it is important to design and evaluate diagnosis strategies that might be used worldwide.

Discrepancies between assays were found. First, three cases of *Rocahepevirus ratti* were identified by the ORF1 assay but not by that targeting ORF3. This decrease in the sensitivity for amplification of RHEV of assays developed for the detection of HEV has also been observed by commercial kits (Behrendt et al., 2021), showing a reduction of approximately 5 log_10_ IU/mL. Recently, the zoonotic potential of RHEV has been recognized, raising the number of cases up to almost 40 on different continents, including chronic infection and two fatal cases (Rivero‐Juarez et al., 2022; Sridhar et al., 2021, 2022). The European Centre for Disease Control (ECDC) noted that RHEV can be an emerging infectious disease, and more sporadic human cases can also be expected in the EU/EEA countries (ECDC, 2022). For this reason, the inclusion of a molecular approach with a tested capacity to detect RHEV in the diagnosis algorithm of HEV is mandatory. On the other hand, there is discrepancy between assays in samples bearing HEV genotype 3f. This genotype is one of the most prevalent worldwide and is highly endemic in Africa and Europe (Casares‐Jimenez et al., 2021). Recently, it has been noted that this genotype presents a high variability, suggesting that it might be divided into three different clades (Muñoz‐Chimeno et al., 2022). This could be the reason for the low sensitivity found in the detection of this genotype during the validation of the World Health Organization International Reference Panel by most of the labs involved in the study (Baylis et al., 2019). In the same way, during the clinical validation of the ORF3 assay, where most sequences were identified as 3f, the two comparator assays used failed to detect up to 30% of patients infected by this genotype (Frías et al., 2021). The ORF3 assay was able to detect 15 individuals infected by genotype 3f not detected by the ORF1 assay, and conversely, using the ORF1 assay, eight additional individuals were detected. This is especially relevant because this genotype has been associated with a more severe disease presentation (Peeters et al., 2023; Schemmerer et al., 2022). For this reason, because of the discrepancy found between assays for genotype 3f, with a significant number of false‐negative individuals, it seems logical to recommend the use of two assays in settings where this genotype is highly prevalent, such as Europe.

Three limitations should be noted for a correct interpretation of our results. First, the study was conducted in a single country with a low variety of HEV genotypes (Muñoz‐Chimeno et al., 2022). Validation of this approach in other settings with different genotypes should be performed. Second, there was a relatively high number of unsequenced samples. For sequencing, we used a standardized protocol employed in HEVnet. Nevertheless, equal to the molecular diagnosis of HEV, typing discrepancies, including sequencing failures, have been found using this assay (Baylis et al., 2022), requiring urgent harmonization and optimization. Finally, one of the assays employed in our study was a nested PCR, limiting the widespread use of the algorithm proposed. Nested PCRs are avoided in the majority of clinical diagnostic labs because the risk of environmental amplicon contamination, and consequently, the risk of false‐positive samples. To facilitate the implementation the algorithm presented here, it will be necessary to optimize a multiplex qPCR combining both methods.

In conclusion, our study found that the parallel use of two broad‐range PCR assays targeting different regions of the viral genome significantly increased the performance of the molecular diagnosis of HEV. This approach should be recommended for the detection of emerging genotypes, particularly in regions where highly divergent genotypes are prevalent.

## AUTHOR CONTRIBUTIONS

**Pedro Lopez‐Lopez:** Data curation (equal); formal analysis (equal); investigation (equal); methodology (equal); software (equal); visualization (equal); writing – original draft (lead); writing – review and editing (equal). **Mario Frias:** Formal analysis (equal); investigation (equal); methodology (equal); resources (equal); writing – review and editing (equal). **Ana Belén Pérez:** Data curation (equal); investigation (equal); methodology (equal); resources (equal); writing – review and editing (equal). **Carolina Freyre‐Carrillo:** Data curation (equal); investigation (equal); resources (equal); writing – review and editing (equal). **Juan A Pineda:** Data curation (equal); investigation (equal); resources (equal); visualization (equal); writing – review and editing (equal). **Antonio Aguilera:** Data curation (equal); investigation (equal); methodology (equal); visualization (equal); writing – review and editing (equal). **Ana Fuentes:** Investigation (equal); methodology (equal); resources (equal); writing – review and editing (equal). **Juan Carlos Alados:** Investigation (equal); methodology (equal); resources (equal); writing – review and editing (equal). **Gabriel Reina:** Investigation (equal); methodology (equal); resources (equal); writing – review and editing (equal). **Encarnacion Ramirez‐Arellano:** Investigation (equal); methodology (equal); resources (equal); writing – review and editing (equal). **Isabel Viciana:** Investigation (equal); methodology (equal); resources (equal); visualization (equal); writing – review and editing (equal). **Joao Mesquita:** Data curation (equal); investigation (equal); methodology (equal); resources (equal); visualization (equal); writing – review and editing (equal). **Javier Caballero‐Gomez:** Data curation (equal); investigation (equal); methodology (equal); resources (equal); visualization (equal); writing – review and editing (equal). **Antonio Rivero‐Juarez:** Conceptualization (equal); data curation (equal); formal analysis (equal); funding acquisition (lead); investigation (equal); methodology (equal); project administration (lead); resources (lead); software (equal); supervision (lead); validation (lead); visualization (lead); writing – original draft (equal); writing – review and editing (equal). **Antonio Rivero:** Conceptualization (equal); funding acquisition (equal); investigation (equal); methodology (equal); resources (equal); supervision (equal); validation (equal); visualization (equal); writing – original draft (equal); writing – review and editing (equal).

## FUNDING INFORMATION

This work was supported by Secretaría General de Investigación, Desarrollo e Innovación en Salud (PI‐0287‐2019) for grants for the financing of Investigación, Desarrollo e Innovación Biomédica y en Ciencias de la Salud en Andalucía; the Ministerio de Sanidad (RD12/0017/0012) integrated into the Plan Nacional de I + D + I and co‐financed by the ISCIII‐Subdirección General de Evaluación and the Fondo Europeo de Desarrollo Regional (FEDER); the Fundación para la Investigación en Salud (FIS) del Instituto Carlos III (Research Project grant numbers: PI19/00864, PI21/00793 and PI22/01098). Antonio Rivero‐Juarez is the recipient of a Miguel Servet Research Contract by the Ministerio de Ciencia, Promoción y Universidades of Spain (CP18/00111). Mario Frias is the recipient of a Sara Borrell Research Contract program by the Ministerio de Ciencia, Promoción y Universidades of Spain (CD18/00091). AR is the beneficiary of Contratos para la intensificación de la actividad investigadora en el Sistema Nacional de Salud by the Ministerio de Ciencia, Promoción y Universidades of Spain (INT20‐00028). Javier Caballero Gómez is supported by the CIBER‐Consorcio Centro de Investigación Biomédica en Red‐(CB21/13/00083), Instituto de Salud Carlos III, Ministerio de Ciencia e Innovación and Unión Europea‐NextGenerationEU.

## CONFLICT OF INTEREST STATEMENT

The authors declare that they have no competing interests. Neither the authors nor their institutions have at any time received payment or services from a third party for any aspect of the submitted work (data monitoring board, study design, manuscript preparation, statistical analysis, and so on).

## Supporting information


Table S1.

## Data Availability

All data generated or analysed during the study are included in the article. The datasets used and/or analysed during the present research project are available from the corresponding author upon reasonable request. All sequences were submitted to GenBank (accession numbers: MN628557 to MN628567, MT250081 to MT250083, MN537838, MN914126, MN914127, MT776550 to MT776554, MT854329, MW143072, OM236461 to OM236465, MZ964415, MZ964416, OL741633 to OL741655, ON720840 to ON720847, ON921072 to ON921078, and OP793784 to OP793790).
