# Supplementary material for: Optimization of the molecular diagnosis of the acute hepatitis E virus infection
Source: Microb Biotechnol. 2023 Mar 25;16(6):1325–32. doi: 10.1111/1751-7915.14247 (PMC10221520; doi:10.1111/1751-7915.14247)
Supplement: Supplementary file 1 — Table S1. [file MBT2-16-1325-s001.docx]

Supplementary Table 1. Primers and probe sets for each PCR reaction.

| **ORF3 PCR assay** | | | | |
| --- | --- | --- | --- | --- |
| Forward (5´-3´) | Reverse (5´-3´) | Probe (5´-3´) | Reference |  |
| RGTRGTTTCTGGGGTGAC | AKGGRTTGGTTGGRTGA | FAM-TGAYTCYCARCCCTTCGC-TAMRA | [19] |  |
| **ORF1 PCR assay** | | | | |
| **First round** | | | | |
| Forward (5´-3´) | Reverse (5´-3´) |  | Reference |  |
| TCGCGCATCACMTTYTTCCARAA | GCCATGTTCCAGACDGTRTTCCA |  | [20] |  |
| **Second round** | | | | |
| Forward (5´-3´) | Reverse (5´-3´) |  | Reference |  |
| TGTGCTCTGTTTGGCCCNTGGTTYCTG | CCAGGCTCACCRGARTGYTTCTTCCA |  | [20] |  |
